# The Pathogenesis of Foot-and-Mouth Disease in Pigs

**DOI:** 10.3389/fvets.2016.00041

**Published:** 2016-05-23

**Authors:** Carolina Stenfeldt, Fayna Diaz-San Segundo, Teresa de los Santos, Luis L. Rodriguez, Jonathan Arzt

**Affiliations:** ^1^Agricultural Research Service (ARS), Foreign Animal Disease Research Unit (FADRU), Plum Island Animal Disease Center (PIADC), United States Department of Agriculture (USDA), Greenport, NY, USA; ^2^PIADC Research Participation Program, Oak Ridge Institute for Science and Education, Oak Ridge, TN, USA; ^3^Department of Pathobiology and Veterinary Science, CANR, University of Connecticut, Storrs, CT, USA

**Keywords:** foot-and-mouth disease, foot-and-mouth disease virus, pigs, pathogenesis, host response, virus diseases, virology

## Abstract

The greatest proportion of foot-and-mouth disease (FMD) clinical research has been dedicated to elucidating pathogenesis and enhancing vaccine protection in cattle with less efforts invested in studies specific to pigs. However, accumulated evidence from FMD outbreaks and experimental investigations suggest that critical components of FMD pathogenesis, immunology, and vaccinology cannot be extrapolated from investigations performed in cattle to explain or to predict outcomes of infection or vaccination in pigs. Furthermore, it has been shown that failure to account for these differences may have substantial consequences when FMD outbreaks occur in areas with dense pig populations. Recent experimental studies have confirmed some aspects of conventional wisdom by demonstrating that pigs are more susceptible to FMD virus (FMDV) infection *via* exposure of the upper gastrointestinal tract (oropharynx) than through inhalation of virus. The infection spreads rapidly within groups of pigs that are housed together, although efficiency of transmission may vary depending on virus strain and exposure intensity. Multiple investigations have demonstrated that physical separation of pigs is sufficient to prevent virus transmission under experimental conditions. Detailed pathogenesis studies have recently demonstrated that specialized epithelium within porcine oropharyngeal tonsils constitute the primary infection sites following simulated natural virus exposure. Furthermore, epithelium of the tonsil of the soft palate supports substantial virus replication during the clinical phase of infection, thus providing large amounts of virus that can be shed into the environment. Due to massive amplification and shedding of virus, acutely infected pigs constitute a considerable source of contagion. FMDV infection results in modulation of several components of the host immune response. The infection is ultimately cleared in association with a strong humoral response and, in contrast to ruminants, there is no subclinical persistence of FMDV in pigs. The aim of this review is to provide an overview of knowledge gained from experimental investigations of FMD pathogenesis, transmission, and host response in pigs. Details of the temporo-anatomic progression of infection are discussed in relation to specific pathogenesis events and the likelihood of transmission. Additionally, relevant aspects of the host immune response are discussed within contexts of conventional and novel intervention strategies of vaccination and immunomodulation.

## Relevance

Foot-and-mouth disease (FMD) is recognized as one of the most contagious and economically important diseases of domestic livestock. The etiological agent, FMD virus (FMDV), an aphthovirus of the *Picornaviridae* family, is capable of infecting a multitude of cloven-hoofed animal species including both ruminants and suids (1, 2). Although domestic cattle are often prioritized with regards to FMD prevention and strategic countermeasures, it is important to recognize that pigs constitute a substantial proportion of agricultural production in large areas of the world. Even though cattle and pigs may be similarly susceptible to FMDV infection under most circumstances, there are critical differences in FMD pathogenesis and infection dynamics that emphasize the importance of species-specific experimental investigations and adaptation of countermeasure policies. Important distinctions between cattle and pigs in FMD pathogenesis events include variations in permissiveness to infection by different routes of virus exposure and thereby differences in the most likely mechanisms of virus transmission between animals. Furthermore, variations in the quantities of virus shed by aerogenous routes, as well as the capability of long-term persistence of infectious virus in tissues of ruminants, but not pigs, indicate important differences pertaining to risk assessments and practical management of infected or convalescent animals.

It is well known that the clinical severity of FMD may vary greatly depending both on the virus strain and the affected host species (1, 2). Acute clinical FMD has been reported to be more severe in pigs compared to ruminant species (1). Contrastingly, pigs are more efficient in complete clearance of the infection, and there is no subclinical “FMDV carrier state” in suids (3). It has also been widely accepted that while pigs are capable of generating large amounts of aerosolized virus, they are less susceptible to airborne infection compared to ruminants (4, 5). Demonstrated variability in host range of certain FMDV strains that are significantly attenuated in cattle, yet virulent in pigs provides additional evidence of the existence of host-specific differences in the molecular pathways of FMDV infection (6–9). Specifically, it was confirmed that a mutation within the FMDV 3A coding region was the determinant for the strictly porcinophilic phenotype of the serotype O FMDV that caused an outbreak in Taiwan in 1997 (8, 10).

A large proportion of experimental studies investigating FMDV pathogenesis and vaccinology have been performed in cattle. Furthermore, the guidelines for FMDV vaccine production published by the World Organization for Animal Health (OIE) only define procedures for efficacy testing in cattle (11). In many regions, it is common practice to vaccinate only cattle, but not pigs, based on the assumption that this practice may be sufficient to prevent dissemination of a potential outbreak. This premise may be misguided if extrapolated to regions with intensive pig production or substantial quantities of wild suids. Several experimental studies have demonstrated difficulty in achieving sufficient protection against clinical FMD in pigs by vaccination, especially when the virus challenge consisted of direct exposure to clinically infected pigs (12–15). Additionally, recent experiences from South Korea have shown that high quality FMDV vaccines with confirmed efficacy in cattle may fail to elicit sufficient levels of immunity (based on serum neutralization testing) when administered to pigs in commercial production settings (16). These distinct, porcinocentric scenarios may be explained by species-specific differences in susceptibility to the virus or by differences in the host response to vaccination. Regardless of the causality, the documented variations between cattle and pigs in outcomes of both vaccination and infection suggest that FMD control policies may, justifiably, be based on species-specific data and should be adapted to account for the composition of the animal population in any given region. Such differences are also highly relevant for disease modeling, wherein it is critical to account for species-specific aspects of FMDV infection dynamics and transmission in order to precisely model distinct scenarios.

## FMD in Pigs

### Routes of Infection

Early experimental studies performed by Terpstra (17) concluded that pigs were highly susceptible to FMDV *via* artificial aerosol exposure, while a 1000-fold higher inoculation dose was required to achieve successful infection by virus instillation in the oral cavity. This was subsequently contradicted in works by Alexandersen and Donaldson which demonstrated that pigs were largely resistant to FMDV infection by inhalation of naturally produced aerosols (4, 5). Additionally, more recent investigations have confirmed that the porcine upper respiratory tract (nasopharynx) is less permissive to inoculation by direct deposition of virus when compared to the upper gastrointestinal tract (oropharynx) (18, 19). Infection *via* the oral route is likely mediated by virus entry through the mucosal surfaces of the oropharyngeal tonsils rather than trough the lower gastrointestinal tract. This is supported by demonstrated tropism of tonsillar epithelium to primary FMDV infection (20) as well as the instability of FMDV at low pH (21, 22), which likely leads to dissociation of virus particles that reach the stomach. The predilection for virus entry *via* the porcine upper gastrointestinal tract is in direct contrast to primary FMDV infection of cattle, which has been demonstrated to occur in the upper respiratory tract (23–26). However, despite this apparent discrepancy in anatomic location, there are striking similarities in microanatomic characteristics of the epithelium that supports primary FMDV replication in both cattle and pigs (19, 23, 25). Specifically, in both species, primary infection occurs at distinct regions of epithelium overlaying mucosa-associated lymphoid tissue (MALT). In these regions (so-called reticular- or follicle-associated epithelium), the epithelium is intimately associated with the subjacent lymphoid follicles, the basement membrane is discontinuous, and there are abundant intraepithelial (transmigrating and resident) leukocytes.

The relative resistance of pigs to aerogenous FMDV infection has been further corroborated by several experimental studies, which have shown that physical separation of pigs is sufficient to prevent transmission of virus under experimental conditions (27–29). Contrastingly, direct contact exposure leads to rapid transmission of infection within groups of pigs that are housed together. Furthermore, it has been demonstrated that this system of virus exposure is often sufficient to overcome vaccine protection (15) even though vaccination may reduce shedding of virus and thereby lower the transmission rate (12, 14). The efficiency of transmission of FMDV under experimental conditions varies between different strains of FMDV (30, 31). Additionally, external factors, such as housing density, the intensity of interactions between animals, and the duration of exposure, will directly influence the outcome of experimental transmission studies (30, 32, 33). Even though these findings strongly suggest that direct physical contact between pigs facilitates FMDV transmission, the specific route of virus entry during contact exposure has not been completely identified. The susceptibility of the porcine oropharyngeal mucosa to FMDV infection would support virus transmission *via* the oral route, e.g., from salivation and subsequent ingestion of shed virus during communal feeding. However, direct entry of virus through skin abrasions and punctures derived from biting or oral entry mediated through direct contact to exposed vesicular lesions on donor animals may also constitute likely transmission routes.

There are many options for challenge systems for FMD experimentation in pigs, which reflect the differences described above. FMDV infection in pigs is often achieved by intraepithelial injection of the heel bulb (27, 34–39). This technique is convenient for vaccine studies, as the pedal epithelium is highly permissive to FMDV infection, leading to substantial amplification of the injected virus at the inoculation site and consistently rapid progression of generalized FMD in susceptible animals. Despite the convenience and consistency of injection-based inoculation techniques, these systems are less appropriate for studies of disease pathogenesis as they are based on an artificial route of virus entry that bypasses the natural barrier of the mucosal immune system. As mentioned above, direct contact exposure to infected animals is highly efficient in generating infection in susceptible animals. However, critical factors, such as the dose and timing of virus challenge, are difficult to control in contact-based systems, which may lead to inconsistencies across studies or misinterpretations of experimental outcomes. Recent studies have demonstrated that controlled exposure of the porcine upper gastrointestinal tract by deposition of virus inoculum in the oropharynx of sedated pigs is highly efficient in generating consistent and synchronous clinical FMD and may thus be considered a valid alternative to the more traditional injection-based challenge systems (18–20).

### Temporo-Anatomic Progression of Infection

#### Primary Infection (Pre-Viremia)

**Figure 1:**
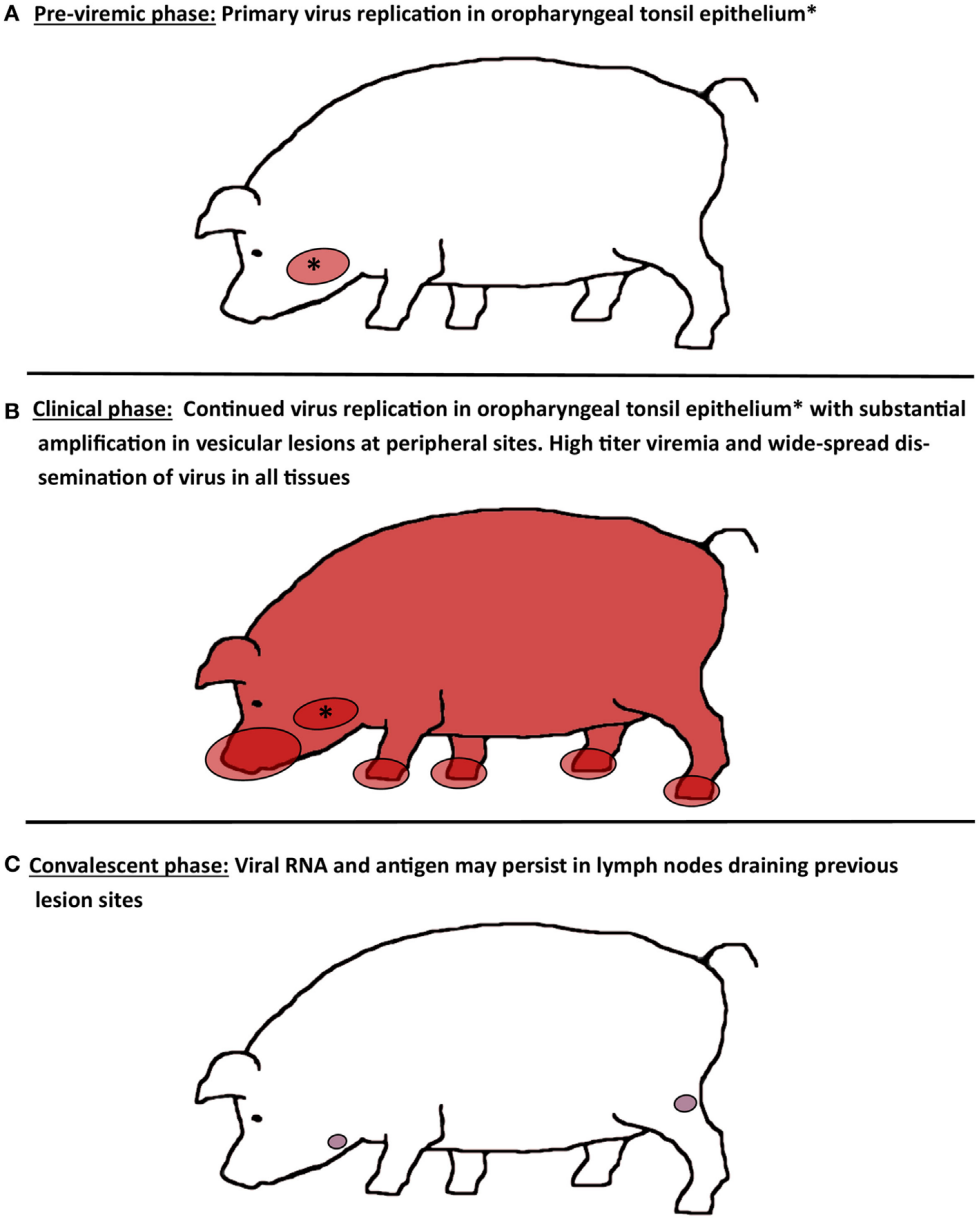
**Schematic illustration of virus distribution in tissues during distinct phases of FMD in pigs**. **(A)** During the pre-viremic phase of infection, primary virus replication is localized to epithelium of oropharyngeal tonsils. **(B)** During the clinical phase of infection, FMDV can be recovered from essentially every tissue or organ sampled due to high titers of virus in blood. Virus replication in oropharyngeal tonsil epithelium continues, while substantial amplification of FMDV occurs in vesicular lesions on the feet, snout, and in the oral cavity. **(C)** After resolution of viremia and clinical disease, FMDV genome and antigen can be recovered from lymph nodes that drain lesion sites for up to 2 months. However, there is no persistence of infectious virus.

Relatively few experimental studies have been dedicated to investigation of the progression of FMDV infection in porcine tissues following natural or simulated natural virus exposure (17, 20, 34, 36, 40). There is general agreement across these investigations that epithelial tissues of the oropharynx constitute the main sites of virus replication during early infection, whereas abundant amplification of virus occurs in vesicular lesions at secondary (peripheral) replication sites (Figure 1). However, there are slight variations among published works regarding the interpretation of the precise events that constitute the initial phase of FMDV infection in pigs.

**Figure 2:**
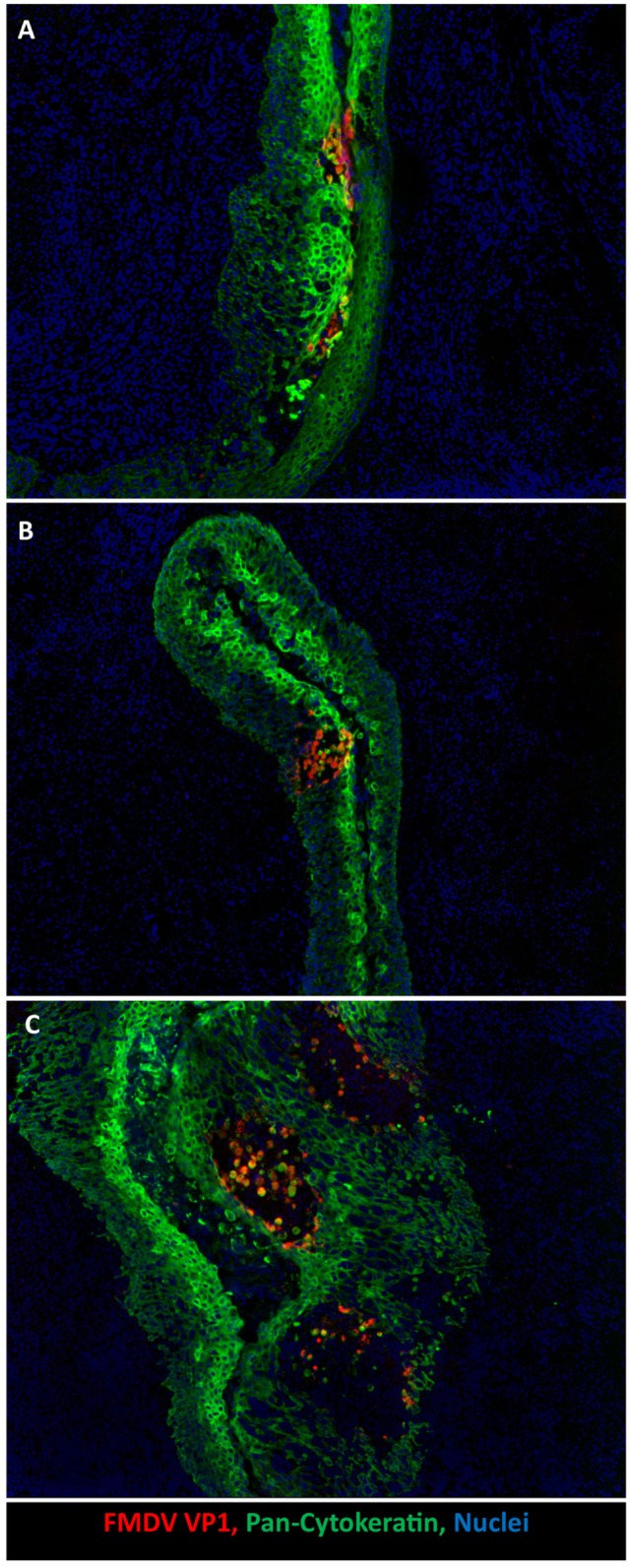
**Development of microvesicles within oropharyngeal tonsil epithelium during early infection**. **(A)** Earliest detection of infection occurs within paraepiglottic tonsil at 24 h post intraoropharyngeal inoculation. FMDV antigen (red) in clusters of infected cytokeratin-positive (green) epithelial cells in superficial layers of crypt epithelium. **(B)** At 48 h post intraoropharyngeal inoculation, a single microvesicle is present within the tonsil of the soft palate. Focus of FMDV-infected (red) epithelial cells expanding through deeper layers of epithelium (green). **(C)** At 78 h post intraoropharyngeal inoculation, three distinct microvesicles are present within crypt epithelium of the tonsil of the soft palate. Sloughed FMDV VP1/cytokeratin double positive cells are present in vesicle lumen. 10× magnification.

A recent investigation demonstrated specific predilection of primary FMDV infection to porcine paraepiglottic tonsils (Figure 2A). This was concluded based on consistent detection of FMDV RNA and infectious virus by qRT-PCR and virus isolation (VI), respectively, prior to the development of viremia and generalization of infection. Additionally, FMDV structural and non-structural viral proteins were localized to crypt epithelium of this specific tonsil by immunomicroscopy at 6–24 h post intraoropharyngeal inoculation (20). Early detection of FMDV RNA and infectious virus was more variable in the tonsil of the soft palate, lingual tonsil, and the dorsal soft palate, suggesting that these sites may also be potential sites of primary infection. A similar investigation performed by Murphy et al. (34) reported detection of FMDV RNA in tonsils, submandibular lymph nodes, spleen, liver, tongue, skin, and pharynx, prior to the detection of viremia. However, the earliest time point for tissue collection in this study was 24 h post contact exposure, which may account for the somewhat wider distribution of viral genome. Additionally, in this study, localization of viral replication was not confirmed by VI or microscopy. Similarly, an earlier investigation by Alexandersen et al. (36) concluded that the highest quantities of FMDV RNA during pre-clinical infection of contact exposed pigs were found in the dorsal soft palate and tonsil (24–48 h post exposure). Noteworthy for these latter two investigations is that the term “tonsil” is not further defined anatomically but may be assumed to represent the tonsil of the soft palate. However, there are multiple distinct tonsils in the porcine oropharynx, including the tonsil of the soft palate, lingual tonsil, and paraepiglottic tonsils (41).

An earlier study by Brown et al. (40) described an investigation of tissue distribution of FMDV RNA by *in situ* hybridization (ISH) in pigs infected by intraepithelial injection, as well as morphological characterization of microscopic lesions associated with the detection of viral genome. This study described widespread dissemination of FMDV genome in the epidermis from 24 to 96 hours post infection (hpi), at sites with or without visible FMDV-associated lesions (40). The study does not include determination of the onset of viremia and systemic dissemination of virus in relation to the time points for tissue collection. However, it is mentioned that pigs euthanized at 24 hpi, corresponding to the earliest time point investigated, were clinically depressed with marked vesicles at the epithelial inoculation sites on the snout and lips. The somewhat different findings between these published studies highlights the differences in experimental outcomes pertaining to experimental design, e.g., inoculation/exposure routes and time points included in the investigation, as well as methods used for virus detection. It is clear that detection of virus genome by qRT-PCR or ISH may lead to different outputs compared to VI or detection of antigen by immunomicroscopy. Combining multiple techniques incurs additional cost and time investment, but ultimately provides a more detailed and substantiated experimental output.

#### Viremia and Clinical Disease

In all *in vivo* studies, the onset of viremia is a critical milestone in FMD pathogenesis, as it accompanies a surge in contagion and predicts the impending clinical syndrome. In pigs, viremia may be detected as early as 24 h after natural or artificial virus exposure, and it is associated with a substantial increase in shedding of infectious virus *via* the oropharyngeal route (3, 20, 30, 42). The onset of clinical FMD, which usually occurs approximately 24 h after detection of viremia, is characterized by fever, loss of appetite, and the appearance of vesicular lesions on feet, snout, and within the oral cavity (20, 30, 34). The initial phase of infection, consisting of the progression from primary, pre-viremic, infection to viremia and clinical disease may be prolonged following exposure to an FMDV strain of reduced virulence, or if exposure conditions are less stringent (e.g., suboptimal exposure route, low challenge dose, or time-limited exposure) (19, 27, 30, 33, 43).

During clinical FMD, the highest quantities of infectious virus are found in vesicular lesions in cornified epithelium of the feet (heel bulbs and coronary bands), on the snout and on the dorsal surface of the tongue (20, 34, 36, 40). It has recently been demonstrated that during clinical disease abundant virus replication occurs in epithelial crypts of the tonsil of the soft palate, and that microscopic vesicular lesions containing large quantities of viral protein can be detected at this site (Figures 2B,C) (20). During peak viremia, FMDV RNA and infectious virus can be recovered from essentially any tissue sampled (Figure 1), but such detection represents intravascular, viremic FMDV rather than regional replication (20).

It is well established that clinically infected pigs release infectious FMDV in exhaled air at quantities substantially greater than cattle (43–46). However, the anatomic source of exhaled virus remains incompletely elucidated. Donaldson and Ferris (47) used an approach of direct air sampling from intact or intubated pigs to evaluate the sources of exhaled virus during different phases of FMD. This demonstrated that infectious FMDV was primarily recovered from the upper respiratory tract during early infection, but that both upper and lower segments of the respiratory tract contributed to exhaled virus during the clinical phase of FMD (47). Unfortunately, there was no collection or analysis of tissue samples in this study, and more detailed conclusions regarding the anatomic sites of released virus are therefore lacking. With the exception of Terpstra (17), tissue-based pathogenesis studies have failed to demonstrate substantial amplification of FMDV in porcine lungs (20, 34, 36).

The tonsil of the soft palate is the only tissue in the respiratory or gastrointestinal tract that has been shown to support substantial levels of FMDV replication (20, 36), and it is therefore the best candidate as the source of aerosolized FMDV derived from pigs. This tonsil is located at the dorsal boundary of the oropharynx and is therefore not within the direct route of exhaled air passing from the lungs through the nasopharynx and nasal cavity. However, the tonsil is anatomically continuous with the dorsal soft palate, and therefore FMDV originating from the tonsil may be aerosolized in the nasopharynx. Additionally, exhalation of air through the oropharynx and mouth, as would occur during vocalization, would pass directly across the surface of the tonsil of the soft palate and facilitate direct aerosolization. Another potential source of airborne virus is secondary resuspension of virus that has been shed into the environment in secretions and sloughed vesicles. However, this would not provide an explanation for the apparently higher quantities of aerogenous virus produced by pigs compared to cattle.

Despite relative resistance to FMDV infection *via* aerogenous exposure (4, 5), clinically infected pigs are a potential source of infection for exposed ruminants due to release of large amounts of aerosolized virus (44). Furthermore, the extent of virus dissemination in porcine tissues during viremia is noteworthy in that pigs and pork harvested during the viremic phase of disease contain massive loads of infectious FMDV. Thus, FMDV-infected pigs constitute a considerable source of contagion during the clinical phase of disease, and movement of live pigs or associated products can have substantial impact on disease spread. These aspects of FMD pathogenesis are of critical importance for the establishment of efficient measures to control outbreaks. Stringent restrictions on movements of animals and animal products, as well as depopulation of infected premises are generally required in order to control dissemination of the disease, regardless of whether emergency vaccination is applied.

#### Clearance of Infection

**Figure 3:**
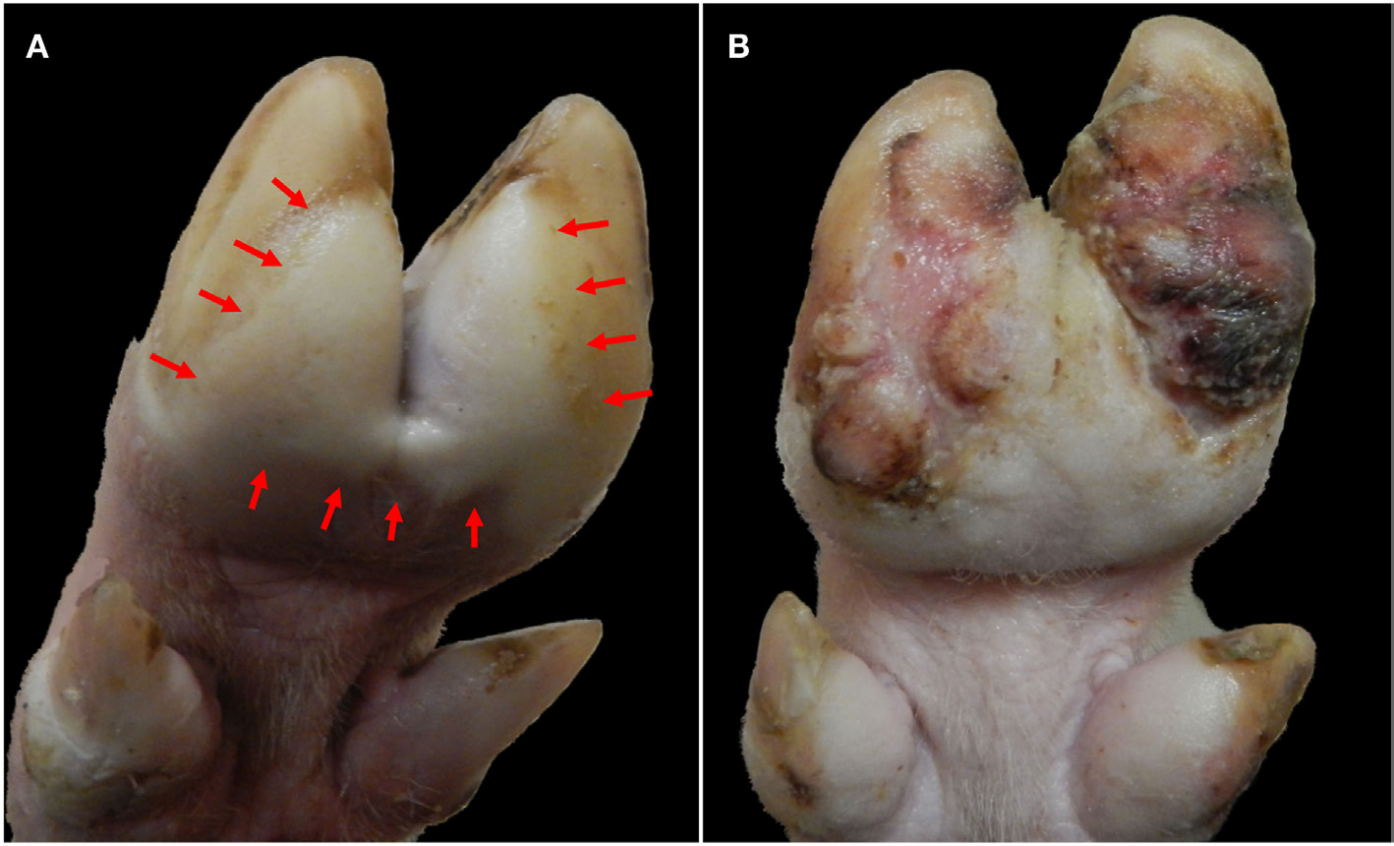
**Progression of foot lesions in pig infected with FMDV A_24_ Cruzeiro at 2 (A) and 24 (B) days after intraoropharyngeal inoculation**. **(A)** Vesicular lesion on solar aspect of hind foot at 2 dpi. Blanching (white) epithelium (delineated within arrows) extends across both heel bulbs and interdigital skin with clear demarcation from normal skin. **(B)** The same animal at 24 dpi. Proliferative dyskeratotic scar tissue has replaced sloughed epithelium.

The clinical phase of FMD subsides within approximately 7–14 days post infection (dpi). In the absence of complications due to secondary bacterial infections, adult pigs generally recover from FMDV infection, although severe foot lesions may cause enduring lameness and debilitation (Figure 3).

FMDV-neutralizing antibodies can be measured in serum of the infected pigs from approximately 4–7 dpi (39, 48). This is followed by a subsequent clearance of infectious virus from blood within approximately 7–14 dpi (3, 49). A single publication by Mezencio et al. (50) concluded that it was possible to detect FMDV RNA in porcine sera as late as 300 dpi. However, this finding has not been repeated or confirmed in any subsequent investigations. Consistent shedding of FMDV RNA can be detected in oral and nasal secretions for up to 14 dpi (3, 49), with some variation across different virus strains. It is likely that the infectiousness of shed virus and thereby contagion associated with pigs recovering from infection is substantially reduced concurrent with increasing titers of neutralizing antibodies in secretions. Nonetheless, despite a large number of published FMDV contact transmission studies, there is no detailed experimental investigation that has precisely documented the duration of infectiousness of FMDV-infected pigs.

In contrast to ruminant species, pigs that survive FMDV infection efficiently clear infectious virus from all tissues after resolution of the clinical disease (3). A study by Rodriguez-Calvo et al. (49) demonstrated that infectious serotype C FMDV could be recovered from porcine tonsils as late as 17 dpi, postulating existence of a putative FMDV carrier state in pigs. A subsequent investigation, using five different strains of FMDV, demonstrated that it was not possible to recover infectious FMDV from any porcine tissues harvested beyond 28 dpi, corresponding to the commonly acknowledged threshold for FMDV persistence (3). However, the same investigation showed that (non-infectious) FMDV RNA could be detected within porcine lymphoid tissue for up to 60 dpi, with highest detection prevalence and most abundant RNA quantities found in the popliteal lymph node that drains the hind feet (Figure 1). Concurrent detection of FMDV structural protein and absence of non-structural protein in popliteal lymph nodes by immunomicroscopy supported the conclusion that viral degradation products may persist in lymphoid organs beyond clearance of infectious virus (3). Detection of FMDV RNA in lymph nodes harvested from both domestic and feral pigs after resolution of clinical disease has been demonstrated in several studies (51–53). However, there is no convincing report documenting isolation of infectious FMDV from porcine tissues beyond 17 dpi.

### FMDV Myocarditis in Pigs

**Figure 4:**
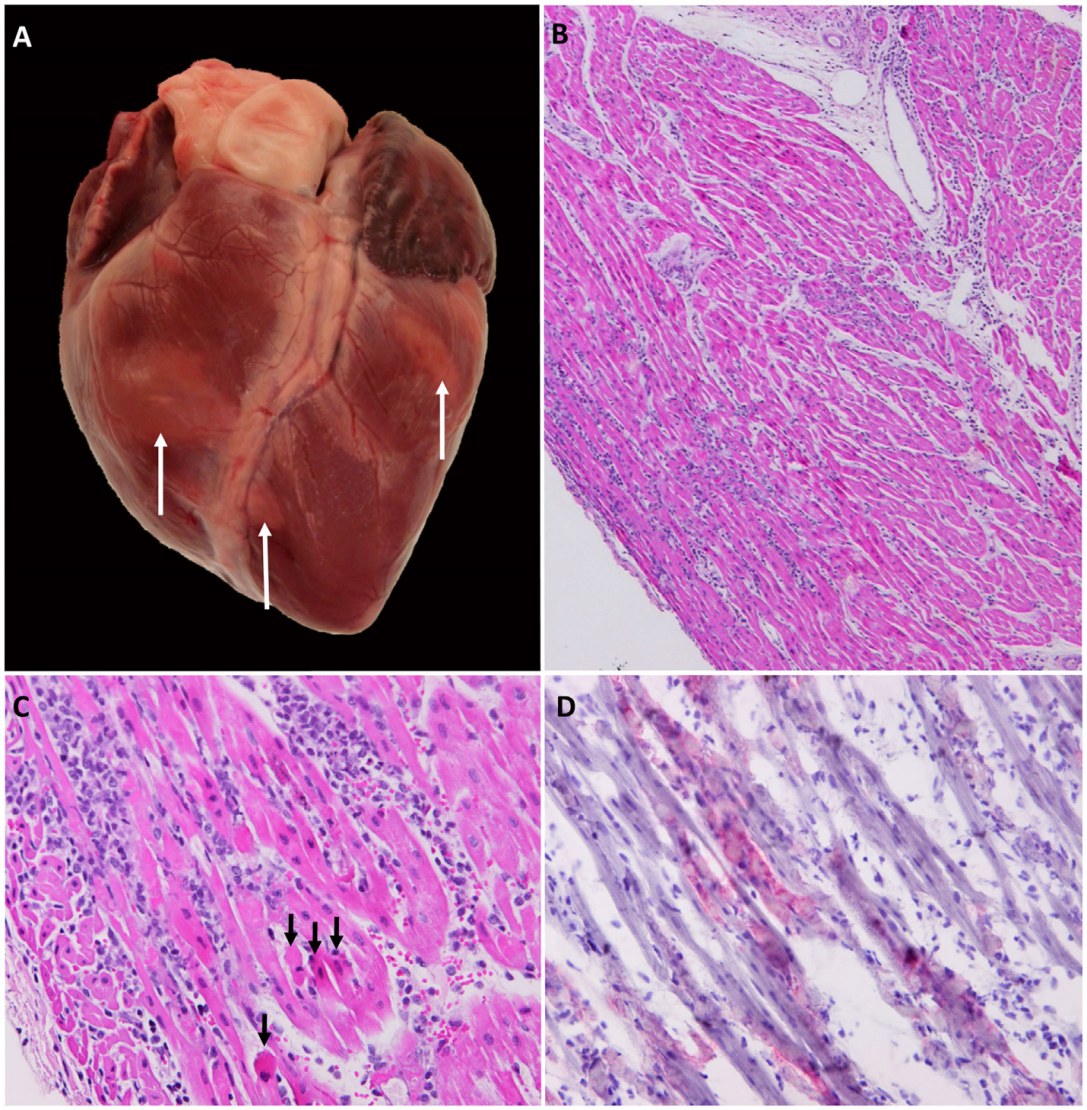
**Gross, histological, and immunomicroscopic characterization of FMDV myocarditis**. **(A)** Gross image of porcine heart with confirmed FMDV-associated myocarditis. Multifocal pallor on the surface of both right and left ventricles (arrows). **(B,C)** Right ventricle of pig found dead at 5 days post infection with FMDV A_24_ Cruzeiro. Interstitial edema and mixed mononuclear infiltrates consisting of lymphocytes, large macrophage-like cells, and scarce neutrophils. Myocyte necrosis and fragmentation (arrows). Hematoxylin and eosin **(B)** 4× magnification, **(C)** 20× magnification. **(D)** Immunohistochemical staining demonstrating localization of FMDV within cardiomyocytes. Anti-FMDV capsid monoclonal antibody (red). Micropolymer alkaline phosphatase. Gill’s hematoxylin counterstain. 20× Magnification. Gross image **(A)** was edited to reduce artifactual glare from flash; lesion areas were not modified.

Although FMD-related mortality among adult pigs is generally low, mortality rates may be higher in juvenile animals (1), and there are often reports of sporadic deaths occurring during experimental studies (6, 19, 31, 54, 55). FMD-related deaths are often attributed to acute viral myocarditis, even in cases when the precise cause of death has not been definitively determined. However, it is well established that acute FMDV infection may cause infection of the myocardium, leading to heart failure and sudden death (56–58). The gross pathological findings associated with FMDV-induced myocarditis may range from complete absence of visible lesions to distinct areas of pallor on the cardiac surface that extend into subjacent myocardium (Figure 4A). Effusion in thoracic and/or abdominal cavities may occur in subacute or chronic cases indicating congestive heart failure, but are often absent in acute, rapidly progressing cases. The terms “tiger stripes” or “tiger heart” are commonly used to describe gross pathological changes associated with FMDV myocarditis. It is our opinion that these are inappropriate and often confusing descriptions as the myocardial pallor induced by FMDV myocarditis rarely assumes a striped pattern. In contrast, stripe-like pale coloration of the myocardial surface is often found as part of normal anatomy associated with superficial vessels and epicardial fat.

Histological findings suggestive of FMDV-induced myocarditis may be predominantly acute and necrotizing or subacute–chronic with various hallmarks of inflammation (Figures 4B,C). Generally, necrosis and inflammation coexist in every lesion with continuum of severity. Inflammation typically includes lymphohistiocytic infiltration and edema, whereas cardiomyocyte degeneration and necrosis may occur as individual cells, small clusters, or may be regionally extensive (Figure 4C) (58). Regions of architectural disruption may have evidence of viral replication including presence of viral antigens and nucleic acids detectable by immunomicroscopy and *in situ* hybridization, respectively (Figure 4D).

Viral loads in the myocardium are massive, often approaching levels otherwise only found in vesicular lesions (58). The prevalence of FMDV myocarditis varies between different strains of FMDV as well as the age of the infected host (2). Interestingly, despite extensive investigation, we have not found evidence of FMDV replication in myocardium of any infected pigs that did not display clinical signs of heart failure.

Since FMDV RNA and infectious virus can be recovered from essentially any tissue harvested during the viremic phase of infection, isolation of FMDV from the heart of a pig with FMD is not sufficient for a diagnosis of FMDV myocarditis. A tentative diagnosis of myocarditis may be based on clinical history of unexpected death in an animal confirmed to be infected with FMD combined with gross findings of (multi)focal mycocardial pallor (Figure 4A). Viral loads in myocardium, which are higher than those detected in serum, provide further support. But, confirmation of FMDV myocarditis requires histopathological identification of lesions combined with immunohistochemical confirmation of presence of FMDV. Additionally, it is noteworthy that identical clinical and histopathological findings have been documented following infection of pigs with encephalomyocarditis virus (EMCV) (59, 60), a related Picornavirus with worldwide distribution (61).

## Host Response to FMDV in Pigs

FMD virus has been demonstrated to modulate the host immune response *via* several mechanisms. Thus, understanding host/pathogen interactions and elucidating the contributions of innate versus adaptive immune responses have become a central topic in FMDV research. Although the host response to FMDV infection is incompletely elucidated, in recent years several studies in swine and cattle have been published, some of them with controversial results. These differences directly reflect the existence of species-specific variations in both systemic and cellular responses to infection (62) that ultimately justifies continued investigation in both suids and ruminants.

### Cellular and Humoral Immune Response

During the early phase of viral infections, interactions between pathogens and cellular components of the innate immune response, such as natural killer (NK) cells, dendritic cells (DCs), and macrophages, define the cellular and humoral adaptive immune response that is believed to ultimately clear the infection. During FMDV replication in pigs, the virus may come in contact with antigen-presenting cells (APCs), either as a result of lytic infection of epithelial cells and subsequent phagocytosis of damaged tissue (63) or by direct infection of immune cells through an antibody-dependent internalization process [macrophages (64, 65) or DCs (66, 67)]. Although the interactions between FMDV and APCs have been shown to be abortive and no virions are produced (63, 67), there are functional consequences that affect the host response. During acute infection, the virus stimulates DCs to produce interleukin (IL)-10, thus directing the adaptive immune response toward a stronger humoral rather than a T-cell-mediated response (67). FMDV also blocks the ability of porcine DCs to differentiate into mature conventional DCs (67) and impairs the response to stimulation by TLR ligands (68).

A distinct subset of DCs; plasmocytoid DCs (pDCs), are susceptible to FMDV infection *in vitro* (66). pDCs internalize immunoglobulin-bound FMDV immune complexes *via* FcγRII surface receptors, and uptake of these complexes results in abortive virus replication (66). Similarly, studies in pigs have shown that these cells are directly affected by FMDV, as the infection leads to depletion of pDCs in peripheral blood, and the remaining pDCs are less capable of producing interferon (IFN)-α in response to *ex vivo* stimulation by TLR ligands or FMDV (69). Porcine Langerhan cells (LCs), identified as a langerin-expressing subset of DCs found in the epidermis (70), are also affected by FMDV infection (68, 71). Although these cells constitutively express type I IFN, *in vitro* studies have demonstrated that FMDV is able to attach to, and become internalized by LCs; however, there is no evidence of internalization leading to replication of viral RNA or production of viral proteins (71). Furthermore, LCs from FMDV-infected pigs produced less IFN-α after *ex vivo* stimulation, although the cells’ ability to present antigen was retained (68).

Natural killer cells also play a critical role during the initial host response to pathogens. Although *in vitro* stimulation of porcine NK cells using pro-inflammatory cytokines induces lysis of FMDV-infected cells and increased expression of IFN-γ (72), *in vivo* studies have demonstrated that NK cells from swine infected with FMDV have a reduced capacity to lyse target cells and secrete IFN-γ (72). NK cell dysfunction during the viremic phase of acute infection suggests that FMDV can effectively block NK function, thereby evading the host’s immune system and promoting virus replication and dissemination within the host.

Another mechanism whereby FMDV may evade the porcine cellular immune response is the induction of severe lymphopenia and lymphoid depletion during peak viremia. The lymphopenia is accompanied by a long-lasting suppression of T-cell function, as T-cells have been shown to respond poorly to mitogen stimulus even after the lymphopenia is resolved (73, 74). However, the mechanisms by which the virus induces this immunosuppression are not completely understood. Lymphocyte depletion and T-cell dysfunction may be caused by viral replication in lymphocytes as has been described during FMDV serotype C infection in swine (73), as well as in *in vitro* experiments investigating FMDV infection of bovine peripheral blood mononuclear cells (PBMCs) (75). However, subsequent studies concluded that active infection of lymphocytes by FMDV could not be demonstrated when pigs were infected with other FMDV serotypes (69, 74). Furthermore, these investigations concluded that FMDV infection was not associated with cell death, suggesting that lymphopenia during FMDV infection might not be related to virus-mediated killing. Additionally, it cannot be ruled out that FMDV-associated lymphopenia may represent a shift of circulating lymphocytes from circulating pool to marginating and tissue-residing pools. An additional mechanism that may contribute to the diminished T cell response during FMDV infection could be related to the elevated amounts of IL-10 produced by conventional DCs that, as mentioned above, has been reported to have immunosuppressive functions *in vivo* for FMDV (67) and for other viruses (76).

Despite the apparent inhibitory actions of FMDV on the cellular host response during early infection, pigs are capable of mounting a substantial humoral response within few days of infection, and there is no documentation of any long-term negative effects on the immune system in pigs that survive FMDV infection. In fact, the high levels of IL-10 during acute infection may skew the adaptive immune response toward a stronger humoral rather than a T-cell-mediated cellular response. The serological response of naive pigs to FMDV infection consists of a rapid surge of anti-FMDV IgM that peaks at 7 dpi and subsequently declines to baseline levels by approximately 4 weeks after infection (39). The IgM response is followed by a sustained anti-FMDV IgG response, which remains at high titers beyond 28 days (39). High titers of neutralizing antibodies can be detected as early as 4–7 days after infection, and unpublished results from our laboratory have confirmed that neutralizing antibody titers remained at high levels as late as 100 days after infection with FMDV A_24_ Cruzeiro. To the best of our knowledge, there is no published documentation of the duration of immunity following FMDV infection in pigs.

### Systemic Antiviral Host Response

Type I, II, and III IFNs, including IFN-α, -β, -γ, and -λ, are critical components of the innate host response to viral infection. Induction of IFN pathways involve initial recognition of pathogen-associated molecular patterns (PAMPs) by cellular pattern-recognizing receptors (PRRs), such as the family of toll-like receptors (TLRs) and cytosolic sensors, eventually leading to the activation of interferon-stimulated genes (ISGs) and production of a variety host proteins with antiviral functions (77–82).

FMD virus has been shown to partially counteract the innate immune response *in vitro* by blocking the expression of IFN (83, 84). Similarly, *in vivo* in pigs, it has been shown that during acute infection FMDV suppresses IFN-α production by skin, myeloid, and plasmacytoid DCs (68, 69, 74). However, it has also been reported that FMDV infection induces a systemic IFN response, which coincides with the onset of viremia (69).

Although the extent of endogenous IFN response in FMDV-infected pigs is incompletely understood, it has been thoroughly documented that FMDV replication in pigs is highly sensitive to the exogenous administration of type I, II, and III IFNs delivered using recombinant vector constructs (83, 85–88). Specifically, pigs pretreated with human adenovirus serotype 5 (Ad5) vectors expressing either porcine IFN-α (Ad5–poIFN-α), porcine IFN-β (Ad5–poIFN-β), porcine IFN-γ (Ad5–poIFN-γ), or porcine IFN-λ (Ad5–poIFN-λ) were efficiently protected against challenge with different FMDV serotypes at 1 day after IFN delivery (37, 88–91) and IFN-induced protection has been demonstrated to last approximately 3–5 days (90). Interestingly, combination of type I and type II IFN results in synergistic anti-FMDV activity *in vivo*; swine inoculated with a combination of Ad5–poIFN-α and Ad5–poIFN-γ, at doses that alone do not protect against FMDV, are completely protected against clinical disease and do not develop viremia (88). More recently, a similar approach using an Ad5 that expressed porcine IFN-α and IFN-γ bicistronically also showed an enhancement of the antiviral activity as compared to Ad5 constructs that only expressed either IFN alone (92). Studies aimed at elucidating the mechanisms by which IFN protects swine against FMD have demonstrated that protection of swine inoculated with Ad5–poIFN-α correlated with recruitment of skin DCs (38), which showed a partial maturation phenotype with increased expression of CD80/86 and decreased phagocytic activity (93).

Administration of Ad5–IFN, type I, II, or III to cattle or pigs leads to induction of numerous genes in association with protection against FMDV (38, 88, 93). However, by directly administering IFN constructs, the natural pathways of interaction of unique viral molecules (or PAMPs) with specific PRRs present in host cells are bypassed. Therefore, to induce a broader, enhanced, and prolonged antiviral response, treatment of animals with various PAMPs could potentially result in a positive feedback induction of additional IFN production (94, 95). Recently, two different strategies have successfully exploited this concept in pigs: (i) the use of double stranded RNA, poly IC, in combination with IFN treatment (96) and (ii) expression of a constitutively active transcription factor, IRF7/3 (5D) fusion protein delivered with the Ad5 vector platform (97).

The value of enhanced understanding of IFN and ISG effects upon FMDV replication in pigs derives from the potential to develop combined-delivery products containing FMDV vaccines and select immunomodulatory constructs. Such products could provide rapid onset and broad protection that could prevent primary virus infection prior to the development of vaccine-induced antibodies. Additionally, enhancement of specific pathways of the innate host response may also serve to strengthen the adaptive immune response, ultimately leading to an overall improved vaccine response.

## Concluding Remarks

To summarize the consensus interpretation of typical FMD pathogenesis in pigs, the majority of experimental investigations suggest that primary FMDV replication during pre-viremic infection occurs in the oropharynx. More detailed investigations have identified epithelial crypts of oropharyngeal tonsils as preferred site of primary infection. Oropharyngeal shedding of virus increases substantially concurrent with the development of viremia, which occurs approximately 24 h prior to appearance of clinical FMD lesions. Abundant quantities of infectious virus can be recovered from essentially all tissues harvested during peak virema, although virus replication in the oropharynx and in vesicular lesions on the feet, snout, and in the mouth constitute the most significant sources of contagion during the clinical phase of disease. FMDV modulates the host immune response and causes severe lymphopenia during acute infection. However, there is a strong humoral immune response, and virus is cleared from circulation within 2 weeks of infection. FMDV RNA and structural antigen may be recovered from lymphoid tissues for several weeks after resolution of the clinical disease, but there is no evidence of the existence of an FMDV carrier state in pigs. These distinct aspects of FMD in pigs should be considered in the development and deployment of response policies and in the modeling of FMD in pigs.

## Author Contributions

CS planned the work and drafted the manuscript. FD drafted the section on host response. TS contributed scientific contents. LR contributed scientific contents. JA planned and coordinated the work and contributed in writing the manuscript. All authors have critically reviewed and revised the manuscript and approved the final product.

## Conflict of Interest Statement

The authors declare that the research was conducted in the absence of any commercial or financial relationships that could be construed as a potential conflict of interest.
